# Real-time monitoring and visualization of the multi-dimensional motion of an anisotropic nanoparticle

**DOI:** 10.1038/srep44167

**Published:** 2017-03-08

**Authors:** Gi-Hyun Go, Seungjin Heo, Jong-Hoi Cho, Yang-Seok Yoo, MinKwan Kim, Chung-Hyun Park, Yong-Hoon Cho

**Affiliations:** 1 Department of Physics, Korea Advanced Institute of Science and Technology (KAIST), Daejeon 34141, Republic of Korea; 2 Graduate School of Nanoscience and Technology, Korea Advanced Institute of Science and Technology (KAIST), Daejeon, 34141, Republic of Korea

## Abstract

As interest in anisotropic particles has increased in various research fields, methods of tracking such particles have become increasingly desirable. Here, we present a new and intuitive method to monitor the Brownian motion of a nanowire, which can construct and visualize multi-dimensional motion of a nanowire confined in an optical trap, using a dual particle tracking system. We measured the isolated angular fluctuations and translational motion of the nanowire in the optical trap, and determined its physical properties, such as stiffness and torque constants, depending on laser power and polarization direction. This has wide implications in nanoscience and nanotechnology with levitated anisotropic nanoparticles.

Anisotropically shaped particles, such as nanowires, have attracted much attention due to their directionally dependent properties. Especially, a preferential alignment of the anisotropic-shaped particles under exerted forces by an external potential or by interaction of the anisotropic particles with the surrounding medium makes them interesting in view of their applications. For instance, because an optically trapped nanowire is oriented along the propagation direction of the laser, it can be used as a probe tip of high resolution photonic force microscopy (PFM) working at aqueous environments^1^,^2^,^3^,^4^. In this case, the large axial size of a nanowire makes trapping stable, while the small cross-sectional area ensures high lateral spatial resolution^2^,^3^. It has been also reported that the orientational coupling between liquid crystal molecules and the surface of a nanowire results in the self-assembly properties^5^,^6^ of a nanowire, which are appealing for possible applications in three-dimensional (3D) photonic crystals^7^ and metamaterials^8^.

However, considering practical applications, there exist specific difficulties involved with precise motion tracking of anisotropic-shaped particles. In general, image information only near the vicinity of a specific image plane is acquired by the detector such as a charge coupled device (CCD) camera or a quadrant photodiode, showing the motion of the nanowire’s cross-section at the monitoring image plane. However, because the motion of the cross-section depends not only translational motion but also rotational motion of the nanowire^9^,^10^, it is unable to distinguish the coupled translational and rotational motions and visualize the real time motion of a nanowire.

To detour this critical problem, some researchers previously tried to restrict the motion of a nanowire in two dimensions intentionally^9^,^11^ or to reduce the rotational motion using high power laser^12^. Up to now, direct visualize the motion of a nanowire was impossible without using these kinds of artificial restrictions, although some trapping parameters could be extracted from a cross-correlation of the signals of a quadrant photodiode^2^. In order to monitor the nanowire tip-sample contact position in PFM and understand how nanowires affect the orientational order of the liquid crystal^13^,^14^, it is important to measure both position and orientation of each individual nanowire in real time without the restrictions.

Here, we present an intuitive method for direct monitoring of the motion of a single nanowire (SNW). Using a fast diffraction pattern image tracking system^15^ in two different image planes, the motion of a nanowire is clearly visualized and hence the translational and rotational motions of the nanowire are separated each other, for the first time to the best of our knowledge. Consequently, optical trapping parameters such as the stiffness and the torque constants of the SNW are determined with varying laser powers and polarizations, which are in good agreement with previous theoretical predictions.

## Results

### Dual image planes monitoring system

**Figure 1:**
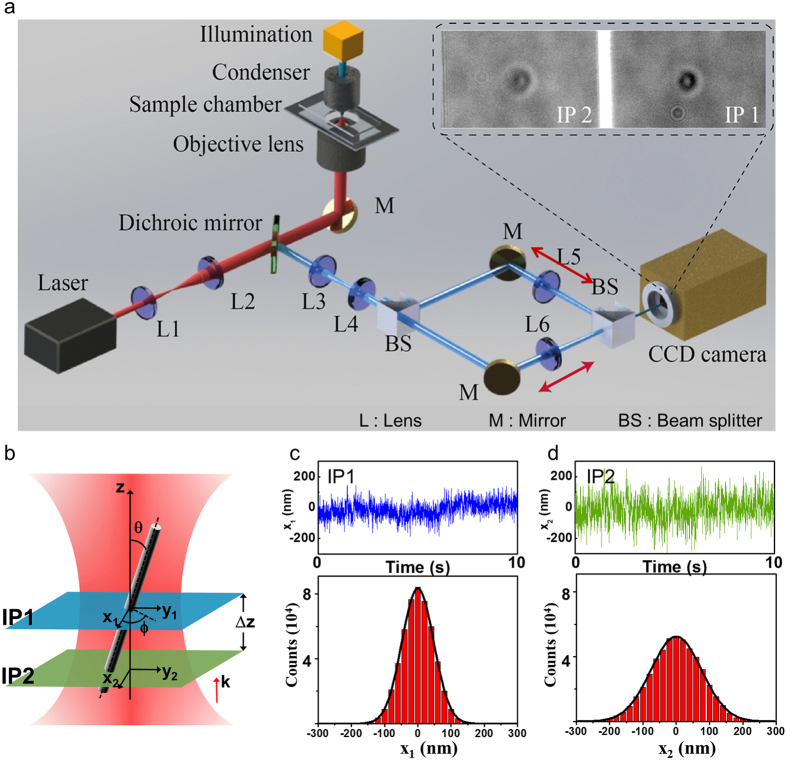
Concept for monitoring the motion of a single nanowire in multi-dimensional directions. (**a**) Optical tweezers setup with real-time dual particle tracking system (the ND filter and the piezo stage are not shown in this figure). The illumination beam on the SNW is propagated through two different paths by the beam splitter. In this case, two different images are projected on the chip of the CCD camera, each in half the total region (Inset image). (**b**) Geometry of the trapped SNW for measuring five degrees of freedom components. The image plane 1 (IP1) and 2 (IP2) can be moved by moving the tube lens L5 and L6. (**c**,**d**) Displacements in the x direction within IP1 (blue) and IP2 (green) plotted vs time and their distributions. The standard deviation of the displacement is 44.7 nm in IP1 and 68.7 nm in IP2.

In order to track the motion of a nanowire in an optical trap, we developed a dual image tracking system, which can simultaneously measure two images at different axial (z-axis) positions (Fig. 1a). A laser (SLOC; IR1064H-800, λ = 1064 nm) which is incident on a high numerical aperture (NA) objective lens is used to trap the suspended gallium nitride (GaN) SNW in the sample chamber. A high power light emitting diode lamp (Thorlabs; M850L3, λ_MAX_ = 850 nm) on top of the microscope illuminates a sample chamber. A beam splitter (BS) separates the light field into two rays, and the customized dual-view setup projects a dual image on the chip area of the CCD camera^16^ (see the inset image in Fig. 1a). Figure 1b shows the geometry of the SNW in the optical trap and two image planes, IP1 and IP2, for measuring *xy* displacements in a specific *z* position, where the trapping position is almost same with the beam waist (Supplementary Information S2) and the position of the image planes can be controlled by moving the tube lens sets, L5 and L6. The IP 1 is fixed at the center of the SNW’s motion (IP1 in Fig. 1b), where the root mean square deviations (RMSDs) of the diffraction pattern is minimum (Fig. 1c). The Brownian fluctuation increases as the position of IP2 in another *z*-axis (IP2 in Fig. 1b) recedes from the position of IP1 because of the increasing contribution of rotational Brownian motion (Fig. 1d). The change in distance of each IP1 and IP2 is calibrated by comparing the different shapes of the diffraction pattern respectively (Supplementary Information S3 and 4).

### Displacement measurement

**Figure 2:**
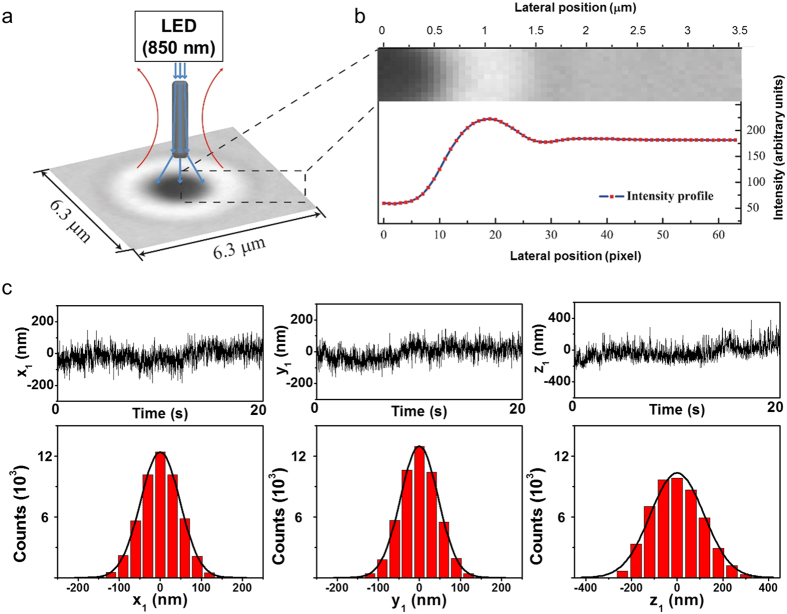
Measuring displacement of a SNW using the image analysis method. (**a**) Schematic of the diffraction pattern imaging of a SNW in the optical trap under LED light illumination (blue line). (**b**) Intensity profile of the diffraction pattern around the center position of the particle. The lateral position can be measured by tracking the center in the diffraction pattern image and the vertical position can be calculated from the intensity profile of the diffraction pattern. (**c**) Displacements of a SNW measured in IP1 and their distributions.

The custom-made particle tracking program measured the position of a trapped SNW with 3D degrees of freedom in the image plane^17^, based on a video image analysis of the trapped particle (see Fig. 2 and the description of the algorithm in the Supplementary Information S3 and 4). We obtained the lateral position (x- and y-axis) of the trapped SNW in the image planes by tracking the center position of the two-dimensional diffraction ring pattern (Fig. 2a) which is shown as a gray-scale image, as recorded by a high-speed complementary metal-oxide-semiconductor (CMOS) camera (Hamamatsu; ORCA-Flash4.0). The vertical position (z-axis) was determined by analyzing the correlation between previous and currently recorded diffraction pattern images (see Supplementary Information S3 and S4). The SNW in the optical trap is aligned in the laser propagating direction because the force exerted on the SNW in the lateral direction is stronger than that exerted in the vertical direction^18^. The large fluctuations of the trapped SNW along the *z*-axis compared with those in the *xy* directions results from the high aspect ratio of the SNW (Fig. 2c)^2^. The RMSDs in *x, y*, and *z* directions were 46.9 nm, 44.7 nm, and 109.8 nm, respectively.

### Coordinate with two image planes

A particle with a simple isotropic shape (e.g., a sphere shaped bead) usually produces isolated 3D Brownian motion in translational directions^19^. In the Brownian dynamics of a SNW, however, the rotational Brownian fluctuations resulting from the anisotropic shape is remarkable^20^,^21^. The contribution of the rotational factor to the Brownian motion of the SNW can be visualized by measuring the lateral displacement of the trapped SNW within two image planes placed at different vertical positions of the trapped SNW. The measured lateral displacement *D*_*i*_ of the trapped SNW in the specific vertical position of SNW is described as follows:


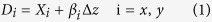


where *X*_*i*_ is the translational displacement and Δ*z* is the gap between IP1 and IP2 (Fig. 1b). *β*_*i*_ is a rotational contribution factor related to the *θ* and *ϕ* angles and is almost zero near the position of IP1. As a result of decoupling the translational and rotational displacements in *D*_*i*_, as described in equation (1), we obtained the *θ* angular displacement, which is the angle between the z- axis and the direction of the trapped SNW and the *ϕ* angular displacement, which represents rotation around the *z*-axis respectively. The angles *θ* and *ϕ* are calculated as follows:


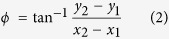



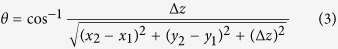


In the experiment, because of the depth of focus, the position of the SNW can slightly different from the position of the diffraction pattern. To compensate this error, the correction term is multiplied by the upper equation, which is calculated by the simulation. (see details in Supplementary Information S5). To confirm the reliability of this description, we measured the angle of the reference sample whose angle can be measured by SEM image. (Supplementary Information S6).

### Shape dependent diffraction pattern

**Figure 3:**
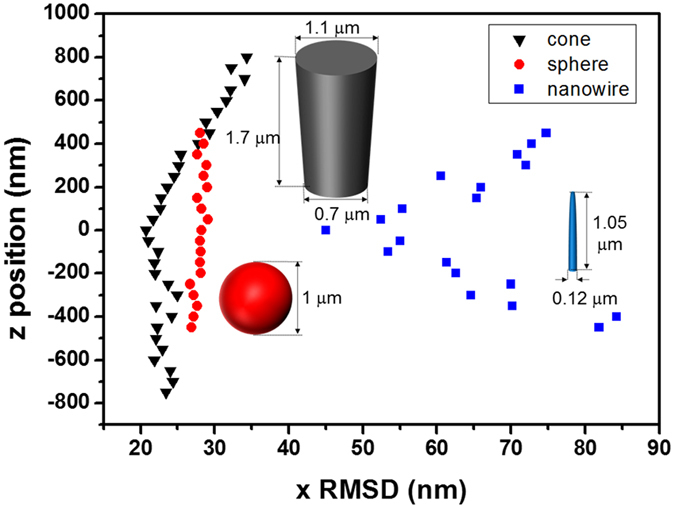
Root mean square deviation in the x direction for different shapes of particles in different movable vertical positions with a 50 nm step. The RMSDs depend on both the shape and the size of the particles. Scale bars in scanning electron microscope images are 1 μm.

To demonstrate that the up-down asymmetry of the trapped particle is related to the rotational Brownian fluctuation, we also measured the RMSDs in the x direction at each vertical position for particles with various shapes, the SNW, a GaN cone shaped particle, and a 1 μm diameter silica sphere bead, as displayed in Fig. 3 (each step is 50 nm). The SNW is considered a rigid rod-like structure with a cylindrical symmetry because the wavelength of the illumination beam is much larger than the SNWs’ diameter (Supplementary Information S7). The SNW shows almost symmetric distribution of x RMSD along the z axis where the z-range for the SNW is 900 nm to ensure that the SNW intersects the image plane (Supplementary Information S8). Meanwhile, the GaN cone-shaped particle, which had an extremely unbalanced ratio of diameters in the top and bottom, showed an asymmetrical RMSD of lateral displacement along the *z*-position. The RMSD of a silica bead, which was almost isotropic in shape, was similar along the whole z-axis, because of the insignificant contribution of rotational Brownian fluctuation to total displacement. On the basis of these results, we confirmed that the up-down asymmetry of the particle in the optical trap resulted in the asymmetry of the distribution of the diffraction pattern along the vertical direction (Supplementary Information S9).

### Visualization of the motion of SNW in three-dimension

**Figure 4:**
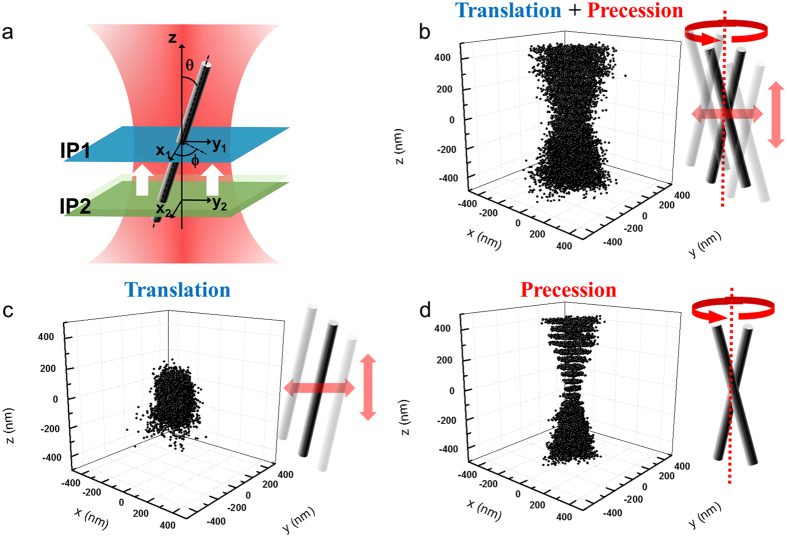
Separation of translation and precession of a SNW in an optical trap. (**a**) Dual image tracking within two image planes. The IP1 is fixed at the center of the SNW motion. The IP2 moves along the vertical direction with a 50-nm step. (**b**) Three-dimensional images of the SNW were constructed by stacking the lateral displacements (*x*_2_, *y*_2_). The displacements result from both the translation and precession of the SNW. From the dual image tracking, the translational motion and the rotational motion of the SNW can be measured, respectively. The translational motions of the SNW’s center are shown in (**c**), and the relative lateral displacements (*x*_2_-*x*_1_, *y*_2_-*y*_1_), which depend only on the precession of the SNW, are shown in (**d**).

To visualize the Brownian motion of the SNW in the optical trap, which rotates around the laser propagating direction and translates in different random directions, we plotted the lateral displacements for the different vertical positions and depicted the results as a 3D image by stacking the plotting data in Fig. 4b. In this case, the displacements showed that the motions of the SNW were composed of translational and rotational Brownian fluctuations within the optical trap. The waist of the 3D stacking graph means the center of the SNW motion. To separate the translational motion and the rotational motion, we conducted the following process. First, we found the center of the SNW motion, where the RMSD had the minimum value, and fixed the image plane 1 (IP 1 in Fig. 4a) at that position. From the dual image tracking we measured the translational motion and the precession of the SNW, respectively. The position of the SNW’s center, (*x*_c_, *y*_c_), can be represented as follows:


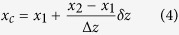



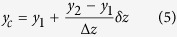


where (*x*_1_, *y*_1_) and (*x*_2_, *y*_2_) are the position of the SNW in the lateral direction, which measured simultaneously within each of the image planes. The Δ*z* is the distance between two different image planes and *δz* is the relative z position measured by analyzing diffraction pattern in IP1 (Supplementary Information S10). The positions of the SNW’s center, (*x*_c_, *y*_c_), which represent the translational motion of the SNW, are shown in Fig. 4c. To monitor the rotational motion of the SNW, we considered the relative coordinate with respect to the center of the SNW motion. The relative displacements can be obtained by subtracting the displacement in IP1 from the displacement in IP2 (in Fig. 4a). Figure 4d shows the relative lateral position along the vertical positions, providing the rotational motion of the SNW solely because the effect of the translational motion is effectively cancelled out.

### Power spectrum analysis for translation

The restoring force exerted on the optically trapped SNW is proportional to the displacement from the trap center, and the trapping potential can be approximated by a harmonic potential with stiffness. We describe the translational Brownian dynamics of the trapped SNW with the Langevin equation as follows:


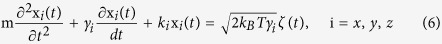


where m is the mass of the particle, *γ* is the friction coefficient, *k* is the stiffness, *k*_*B*_ is the Boltzmann constant, *T* is the temperature, and *ζ(t*) is a stochastic function to represent Brownian fluctuations. The right-hand side indicates the Brownian force acting on the trapped SNW in the aqueous medium. The second-order differential term in equation (6) can be neglected because the momentum relaxation time ( = m/γ ~ 10^−8^ s) is much shorter than our experimental time resolution (10^−3^ s) at a 512 Hz sampling rate.

We use the power spectrum of the translational motion, *P*_t_, to obtain stiffness by taking the Fourier transformation of the Langevin equation^22^. *P*_t_ is given by the Lorentzian form (Supplementary Information S11)


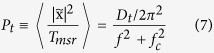


where 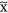 is the Fourier transform of the displacement *x*(t) for the measured time *T*_msr_, 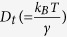 is the diffusion constant for the stochastic variable *x*(t), and 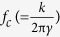 is the corner frequency. The *γ* can be obtained from the geometrical quantities of the nanowire and the viscosity of the solution (see Supplementary Information S12)^23^. For our nanowire, the *γ* value is 4.57 × 10^−6^ g/s in the lateral direction and 3.74 × 10^−6^ g/s in the vertical direction.

**Figure 5:**
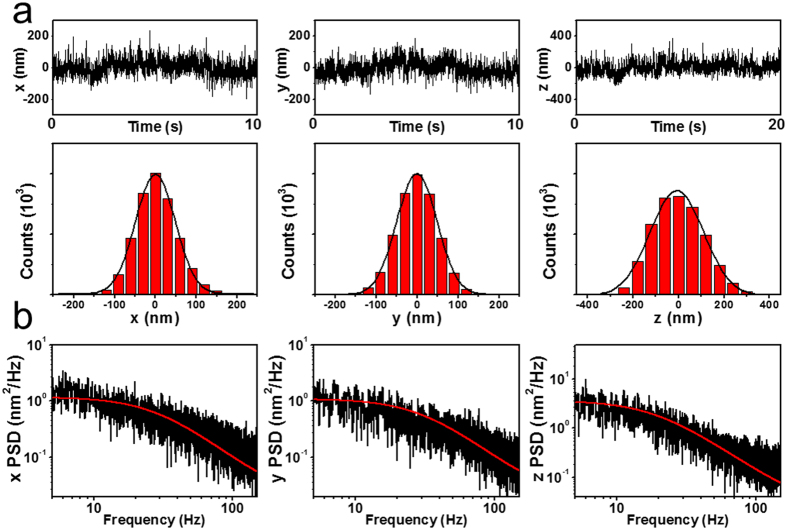
(**a**) Displacements and their distributions in *x, y* and z directions. The RMSD is 97.70 nm for x, 98.70 nm for y, and 234 nm for z. (**b**) Power spectrum of the translational motion acquired in the Fourier domain in *x, y*, and *z* with Lorentzian fitting. The corner frequency is 28.8 ± 0.2 Hz in the *x*-axis, 30.5 ± 0.2 Hz in the *y*-axis, and 19.0 ± 0.1 Hz in the z-axis.

In the practical experiment, the data acquisition rate is a finite value, which results in the aliasing effect of the power spectrum. Due to the effect, the power spectrum is modified to the aliased Lorentzian as shown below^22^,^24^:


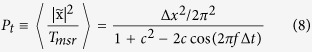


where c ≡ exp(−πf_c_/f_Nyq_), f_Nyq_ ≡ f_Sample_/2 = 256, Δx ≡ ((1 − c^2^)D/(2πf_c_))^1/2^, Δt ≡ 1/f_Sample_, and f_Sample_ is the data acquisition rate. We obtained the power spectrum by averaging from 5 data set. In each data set, we measured the motion of the nanowire for 20 s by using CMOS camera (f_sample_ = 512 Hz). Consequently, we obtained *f*_*c*_ by the aliased Lorentzian fitting in the power spectrum to calculate *k* (Fig. 5). Note that when f_c_ is efficiently small than the f_Nyq_, the aliased Lorentzian becomes the normal Lorentzian.

### Power spectrum analysis for rotation

**Figure 6:**
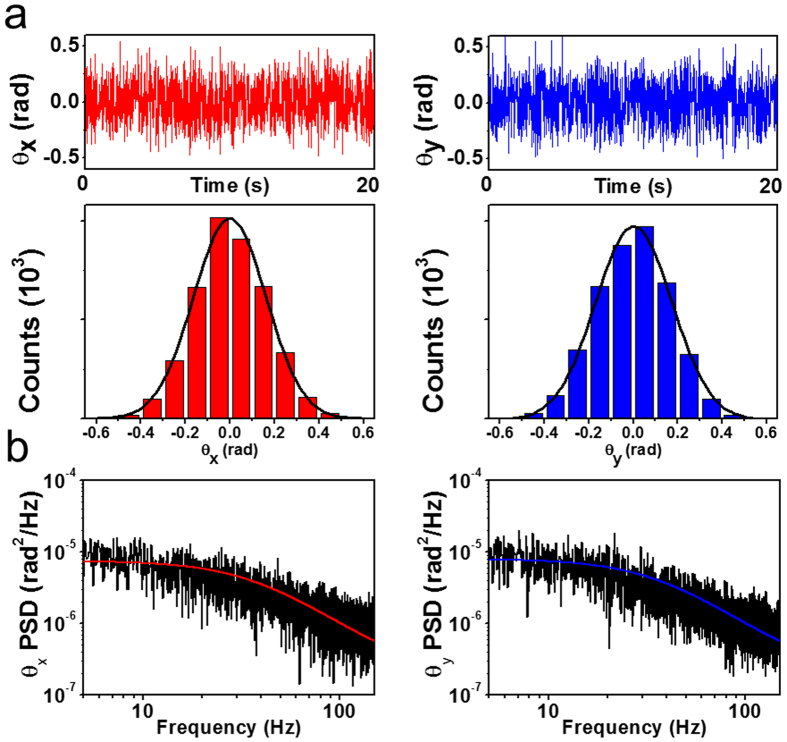
(**a**) Angular displacements and their distributions in *x* and *y* directions. The RMSD is 0.327 rad for θ_*x*_ and 0.345 rad for θ_*y*_. (**b**) Power spectrum of the angular displacement acquired in the Fourier domain in θ_*x*_ and θ_*y*_ with Lorentzian fitting. The corner frequency is 36.9 ± 0.2 Hz in the *x*-axis and 35.4 ± 0.2 Hz in the *y*-axis.

As the SNW has an elongated cylindrical shape, another significant optical trapping parameter, the torque constant, should be considered in the trapped SNW. Torque is proportional to the angle between the direction of the SNW length and the direction of laser propagation. We separate the angles *θ* and *ϕ* as described as θ_*x*_ = *θ* cos *ϕ* and θ_*y*_ = *θ* sin *ϕ* by projection on the *x*- and *y*-axis, respectively. The angular displacements, θ_*x*_ and θ_*y*_, are uncorrelated in our experiment condition, sin *θ* ≈ *θ* (see Supplementary Information S13 and 14). In this case, the angular displacements can be considered as the stochastic random variable, and their distribution can be fitted to the Gaussian (Fig. 6a). Thus we described this stochastic motion by a modified Langevin equation in a form using the angular displacement θ_*i*_(*t*)^25^


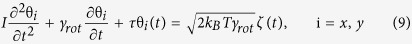


where *I* is the moment of inertia, *γ*_*rot*_ is the rotational friction coefficient^19^, and *τ* is the torque constant. The angular behavior of Brownian motion depends on length, dielectric properties and dominantly, the aspect ratio of the trapped particles. The right-hand side of equation (9) presents the rotational Brownian fluctuation as a result of random torque.

The torque constant is also obtained by the power spectrum, similar to that for stiffness. The result of the Fourier transformation of the equation is


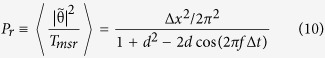


where 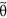 is the Fourier transform of θ(*t*) for time T_msr_, d ≡ exp(−π*ω*_*c*_/f_Nyq_), Δx ≡ ((1 − d^2^)D_r_/(2π*ω*_*c*_))^1/2^, and 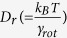 is the diffusion constant for the stochastic variable θ(*t*) and 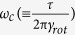 is the corner frequency of the *P*_*r*_. The rotational friction coefficient, *γ*_*rot*_ is determined by the length, shape of the trapped material and the liquid viscosity^23^. For our nanowire, *γ*_*rot*_ is 0.75 × 10^−6^ g μm^2^/s. We can obtain *ω*_*c*_ to obtain the torque constant τ by a Lorentzian fitting in the power spectrum in the *x* and *y* directions, as shown in Fig. 6b. The range of the corner frequency, which is required to obtain the optical trapping parameters of the trapped particle, is within the capabilities of a high speed CMOS camera^26^,^27^.

### Laser dependence of the optical trapping parameter

**Figure 7:**
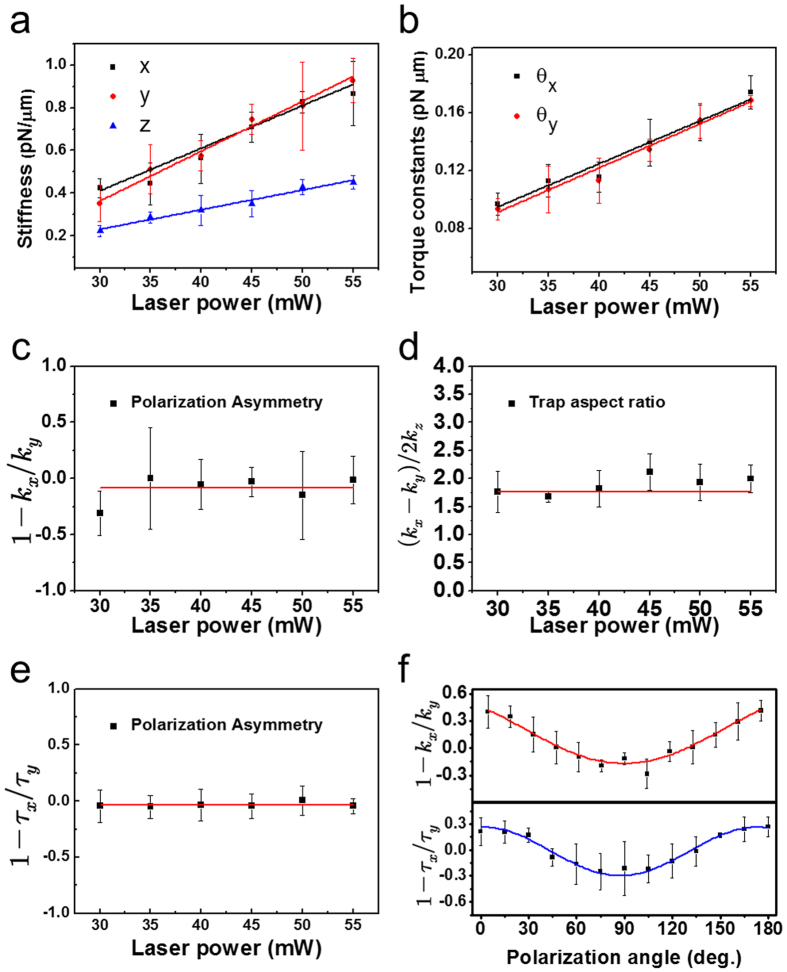
Measurement of mechanical properties of the trapped SNW. (**a**) Stiffness in translational direction (*x, y* and *z*) and (**b**) torque constant in the *x* and *y* direction of laser power variation. (**c**) Polarization asymmetry of *k*_*x*_ and *k*_*y*_, (**d**) Trap aspect ratio of stiffness *k*_*x*_, *k*_*y*_ and *k*_*z*_ and (**e**) Polarization asymmetry of the torque constant. The range of incident laser power is 30–55 mW of the experiments in (**a**–**e**). (**f**) Simultaneously measured polarization asymmetry of stiffness and torque constant, dependent on the polarization angle of the incident laser (0–180°).

To characterize the motion of the trapped SNW, we measured the stiffness in the *x, y*, and *z* direction and the torque constant in the *x* and *y* directions with varying laser power (Fig. 7a and b). We confirmed that the optical trapping parameters, stiffness and torque constant, are proportional to the laser power. The asymmetry polarization effect can be quantified using the parameter, *k*_*p*_ = 1 − *k*_*x*_/*k*_*y*_. As shown in Fig. 7c, we determined a mean value of *k*_*p*_ ≈ −0.09 within the range of the incident laser power with the polarization angle of 45 degrees. Because the laser provides the same potential in the *x* and *y* directions, the value *k*_*p*_ can only be introduced from the geometry of the sample, which exhibits cylindrical symmetry and therefore results in *k*_*p*_ being approximately zero. Additionally, we obtained the trap aspect ratio: *k*_*r*_ = (*k*_*x*_ + *k*_*y*_)/2*k*_*z*_ which shows the characteristics of the particle in the optical trap (Fig. 7d) and is dependent on the size and the asymmetry of the particle. The mean value was found to be *k*_*r*_ ≈ 1.79.

The torque constant is also affected by the polarization asymmetry. We calculated the polarization asymmetry of the torque constant: *τ*_*p*_ = 1 − *τ*_*x*_/*τ*_*y*_ and the mean value was *τ*_*p*_ ≈ −0.05 as shown in Fig. 7e. We measured *k*_*p*_ and *τ*_*p*_ by varying the polarization direction to investigate the polarization asymmetry effect more precisely. The periodicity of *k*_*p*_ and *τ*_*p*_ is evident in Fig. 7f, showing oscillatory variation in the polarization asymmetry as a function of polarization angle. The orientation of the largest trap stiffness and torque constant in the optical trap is perpendicular to the laser’s polarization direction. A linearly polarized single Gaussian beam affects the asymmetric intensity distribution in the *xy* plane in the range of R < *λ*; the sub-micrometer diameter of the SNW was much smaller than the wavelength of the trapping laser (R ≈ 200 nm in our experiment)^18^. The effects of the linear polarized laser on the nanometer scale particles yielded different stiffness values in the *x* and *y* directions^28^. Therefore, our experiment data confirms that we successfully measured the motion of the SNW.

## Discussion

In summary, we described a novel method to accurately observe and visualize the Brownian dynamics of an optically trapped uniaxial object. Our *in situ* real time monitoring system enabled the decoupled measurement of translational and rotational fluctuations using a dual particle tracking system. Determining the position of the trapped SNW is based on a highly sensitive analysis of diffraction pattern images, with nanometer resolution. Consequently, we determined the significant optical trapping parameters such as stiffness and torque constants acting on the SNW in the optical trap, and confirmed that they are proportional to the trapping laser power and show sinusoidal polarization dependence. As the first direct measurement of anisotropic particles, this tracking method with multiple image planes at different axial positions can easily be used to observe the microstates of colloids for studying self-assembly properties, and to develop a high resolution PFM system using the SNW as a scanning probe tip. These have wide applications in physics, materials science, and biotechnology.

## Methods

### Sample preparation

GaN nanowires were obtained by a top-down chemical etching method. For the etching process, we prepared a GaN film grown on a *c*-plane sapphire substrate by metal-organic chemical vapor deposition, and then single crystalline GaN nanowires were formed by a chemical vapor-phase etching process^29^. The lengths of the nanowires are controlled by the thickness of GaN, and the GaN nanowires were mechanically scratched and dispersed in water to obtain SNWs.

### Setup

Optical tweezers were built on a custom-made inverted microscope with optical components and a high numerical aperture (NA) objective lens (Olympus; Plan APO 100×, NA = 1.4) mounted on a vibration-isolating optical table to minimize external noise [sketched in Fig. 1a of the setup]. We used a single-mode, continuous-wave Nd:YAG laser (SLOC; IR1064H-800, λ = 1064 nm) with 2.5 W maximum power as the trapping source. The laser intensity entering the objective lens is controlled by a neutral density (ND) filter to make a bigger gradient force than the scattering force exerted on the SNW, to optimizing the stable trap^30^. To increase trapping efficiency, the size of the laser beam was expanded threefold by collimated lens sets, L1 and L2, to overfill the objective lens aperture^31^. As the expanded laser beam is incident on the objective lens, a highly focused laser in the sample chamber makes a parabolic potential well that prohibits the SNW from escaping the optical trap. A high power light emitting diode lamp (Thorlabs; M850L3, λ_MAX_ = 850 nm, Supplementary Information S15) on top of the microscope illuminates a sample chamber, where light is focused by a condenser (Olympus; Plan APO 10× , NA = 0.55). For stable trapping of the SNW, we made a closed sample cell by sandwiching two cover glasses with double-sided masking tape and injected the solution including the nanowires and sealed them with vacuum grease. The cover glass was washed in a sonicator with potassium hydroxide for 20 min to remove organic dust and dried with 99.9% nitrogen gas. To bring the suspended SNW near the laser focus, we changed the position of the sample chamber by using a micro positioner on the sample stage.

### Simultaneous dual image planes monitoring

The coupling displacement including translational and rotational Brownian motion should be separated to obtain the angular displacement in isolation. We considered the Euler angle (*θ, ϕ, ψ*) of the SNW in the optical trap with respect to a Cartesian coordinate *xyz* axis. The geometry of the trapped SNW in five-dimensional coordinates and two image planes for measuring *xy* displacements in a specific *z*-axis position is shown in Fig. 1b. The image plane 1 is fixed at the center of the SNW (IP1 in Fig. 1b), where the RMSDs of the diffraction pattern is minimum (Fig. 1c). The Brownian fluctuation measured in IP1 indicates the translational displacement. The Brownian fluctuation increases as the position of image plane 2 in another *z*-axis (IP2 in Fig. 1b) recedes from the position of IP1 because of the increasing contribution of rotational Brownian motion (Fig. 1d). The position of IP2 moves along the z-axis of the trapped SNW by scanning the tube lens (L6 in Fig. 1a) back and forth. A beam splitter (BS) separated the light field into two rays, and the customized dual-view setup projects dual images on the chip area of the CCD camera. In this case, IP1 and IP2 are each projected onto half the region of the chip of the CCD camera in order to simultaneously measure their displacements^16^. The change in distance of the IP2 in the trapped SNW is calibrated using a piezo stage on the objective lens. The relationship between the moving distance of the tube lens and the image plane is calculated by comparing the different shapes of diffraction pattern.

## Additional Information

**How to cite this article:** Go, G.-H. *et al*. Real-time monitoring and visualization of the multi-dimensional motion of an anisotropic nanoparticle. *Sci. Rep.* **7**, 44167; doi: 10.1038/srep44167 (2017).

**Publisher's note:** Springer Nature remains neutral with regard to jurisdictional claims in published maps and institutional affiliations.

## Supplementary Material

Supplementary Information
