# Supplementary material for: Real-time monitoring and visualization of the multi-dimensional motion of an anisotropic nanoparticle
Source: Sci Rep. 2017 Mar 8;7:44167. doi: 10.1038/srep44167 (PMC5341161; doi:10.1038/srep44167)
Supplement: Supplementary Information [file srep44167-s1.pdf]

# Supplementary Information for

# Real-time monitoring and visualization of the multi-dimensional motion of an anisotropic nanoparticle

Gi-Hyun Go[1,3], Seungjin Heo[2,3], Jong-Hoi Cho[1], Yang-Seok Yoo[1], Min-Kwan Kim[2], Chung-Hyun Park[1] and Yong-Hoon Cho[1,*]

[1]Department of Physics, Korea Advanced Institute of Science and Technology (KAIST), Daejeon 34141, Republic of Korea

[2]Graduate School of Nanoscience and Technology, Korea Advanced Institute of Science and Technology (KAIST), Daejeon, 34141, Republic of Korea

[3]These authors contributed equally to this work.

Correspondence should be addressed to Y.-H.C (yhc@kaist.ac.kr)

**S1. The preferential alignment of the optically trapped nanowire**

To verify the direction of the single nanowire in the optical volume, we obtained a snapshot in the sample chamber. The laser underwent repeated on- and off- states. The untrapped SNW showed various motions; for example, horizontal or perpendicular to the laser propagating direction. The trapped SNW, however presents a very stable shape of diffraction pattern, where the intensity profile is the same within all radial directions. This result shows that the SNW is trapped in the laser propagating direction.

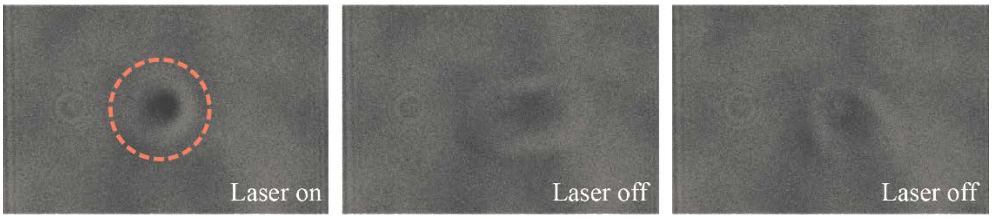

**S2. The gradient force and the scattering force**

Therefore, the scattering force or the radiation force should be considered. Therefore, we checked the effect of both forces for the chain of nine GaN spheres with the diameter of 120nm and the length of 1.08 μm which is similar to our GaN nanowire with a diameter of 120 nm and the length of 1.05 μm.

We calculate the effect of both forces for the chain of nine GaN spheres by following method.

First and foremost, we calculate the gradient force and the scattering force for each spheres as shown below:

The gradient force $F_G = \frac{1}{2}\frac{\alpha}{c\varepsilon_W n_W}\nabla \mathrm{I(r)}$ (S1)

The scattering force $F_S = \frac{k^4\alpha^2}{6\pi c\varepsilon_W^2 n_W^3}\mathrm{I(r)}\hat{\mathrm{z}}$ (S2)

$$\alpha = \frac{4\pi\varepsilon_W n_W^2 a^3(\left(\frac{n_G}{n_W}\right)^2 - 1)}{\left(\frac{n_G}{n_W}\right)^2 + 2} \quad \text{(S3)}$$

where $F_G$ is the gradient force, $F_S$ is the scattering force, $\varepsilon_w$ is the permittivity of the water, $n_G$ is the refractive index of the GaN, $n_w$ is the refractive index of the water, c is the speed of the light, a is the radius of the GaN sphere.

Here we consider the case that the chain does not tilt so that the central axis is equal to the laser propagation direction. In this case, the force in lateral direction can be ignored because the lateral force always cancels out the other lateral force with same magnitude and opposite direction due to its symmetry. Therefore, it’s enough to know the intensity distribution along z direction which described as follow:

$$I(z) = I_0 \frac{z_R^2}{z_R^2+z^2} \quad (S4)$$

where the half of the depth of focus $z_R$ is 359.9 nm for NA=1.4 and λ=1064 nm.

Consequently, we obtained the net force for the chain of the spheres by summation the forces for each spheres. The results are shown in the following figure where we define the z direction or laser propagation direction as positive.

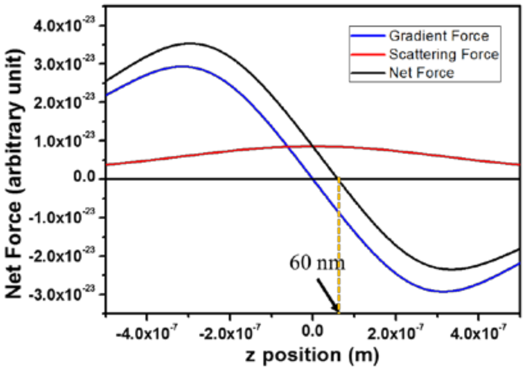


The trapping position is 60 nm where the magnitude of the gradient force is same with the magnitude of the scattering force. Note that we calculate the Rayleigh scattering regime so that the scattering force is overestimated and the trapping position is large compare to the practical case. From these result, we can assume that the gradient force of our GaN nanowire with the diameter of 120 nm is much larger than the scattering force so that the trapping position is not far from the focal position as shown in the Fig 1b.

**S3. Diffraction pattern analysis for tracking the vertical displacements of the SNW**

We chose monochromatic light as the illuminating source for particle tracking because the contrast in the diffraction pattern, which consisted of concentric and alternating light and dark rings, needed to be improved.[1] We also controlled the power of the illuminating light to minimize the effect of the higher wave number sinusoidal function, which contributes to the tracking image, for the accuracy of particle positioning.

To track the displacement in the vertical direction in the SNW, we calculated the correlation between the current and initial images because the fluctuation in SNW causes a change in diffraction pattern shape.[2] Considering that the intensity profile of the diffraction pattern is a linear combination of sinusoidal functions with different wavenumbers, the intensity profile at a given position z in the nanowire follows the equation;

$$I(z) = \sum_{i=0}^{m} A_i \cos[k_i r + \Phi_i(z)] \quad \text{(S5)}$$

where $A_i$ and $\phi_i$ are the amplitude of the light and the phase of the cosine function with wavenumber $k_i$, respectively. To simplify the variation of intensity profile of the radial diffraction pattern, we calculated the characteristic phase difference, $\Delta\Phi(z)$ defined as follows

$$\Phi_z = \Phi(z)_1 + \Phi(z)_2 + \cdots \Phi(z)_m = \sum_{i=n}^{m} \Phi(z)_i \quad \text{(S6)}$$

$$\Delta\Phi(\mathrm{z}) = \Phi(\mathrm{z}) - \Phi(0) \quad \text{(S7)}$$

where $\Phi(z)$ is the current measured characteristic phase, $\Phi(0)$ is the initial measured characteristic and $\Delta\Phi(z)$ is variable with the position between the nanowire and the image plane and is calibrated in distance units.

**S4. Relationship between the changing distance of the tube lens and the image planes**

To acquire the changing distance of the image plane in the sample chamber by scanning the tube lens, we measure delta phase, $\Delta\Phi$ which is a parameter acquired in the diffraction pattern (refer to Supplementary Information 3). First, we measured the $\Delta\Phi$ moving the piezo stage, which is the real changing distance of the image plane. The graph shows the relationship between the changing distance of the tube lens and the piezo stage as a function of $\Delta\Phi$, and we calibrated these two results.

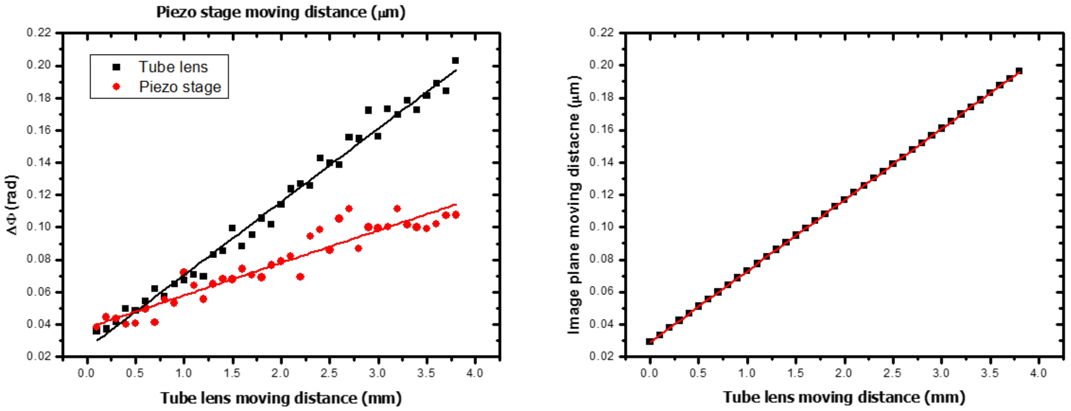

**S5. The effect caused by the depth of focus**

The factors such as LED spectrum and the depth of focus (DOF) should be considered. Therefore, we confirmed their effects by simulation as shown below. First we calculate the point spread function (PSF) of the field $h_z(x,y)$ in the vicinity of the image. Because the width of the nanowire is much smaller than the LED wavelength, we can regard the nanowire as the chain of the point sources. Then the PSF of the field with considering the DOF and the slope of nanowire is just the summation of the shifted PSF of the field at each vertical position (see figure below). After summation, the point spread function of the intensity, the absolute square of the PSF of the field, can be obtained.

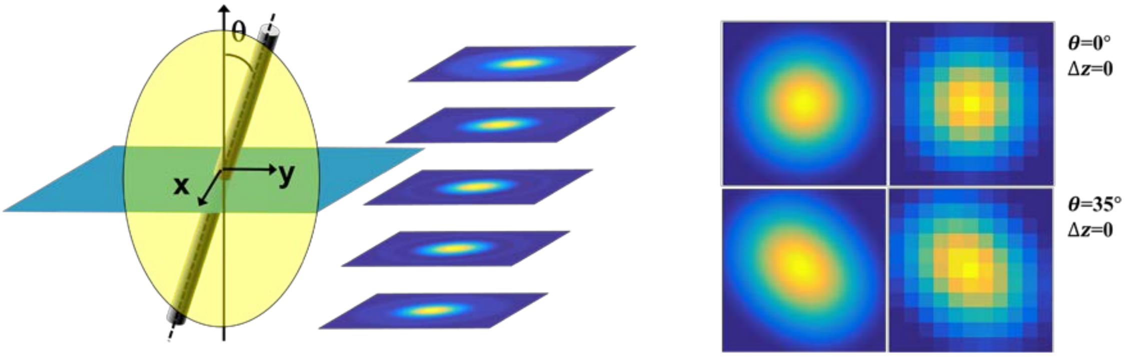


To consider the LED spectrum and the CMOS camera quantum efficiency, we integrate the PSFs of the intensity for various wavelength with multiplying weight factor, which was obtained by multiplying LED spectrum and CMOS camera quantum efficiency. Because of the DOF, the images result from both the slope of the nanowire and the relative position of the image plane.

The effect of image plane positions is shown below:

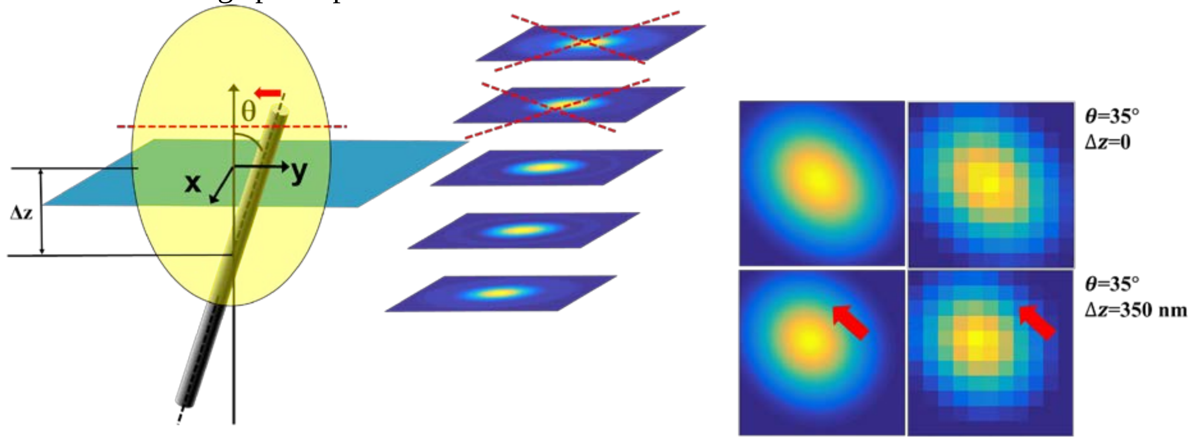


As the position of the image plane recede from the center of the nanowire, the part of the PSF is omitted due to the absence of the nanowire. Because of the omission of the PSF, the angle is underestimated (see red arrow in the upper figure).

We track the center of the image by using tracking algorithm with varying the nanowire angles and the vertical positions of the image plane from 50 nm to 450 nm. The results are shown below.

.

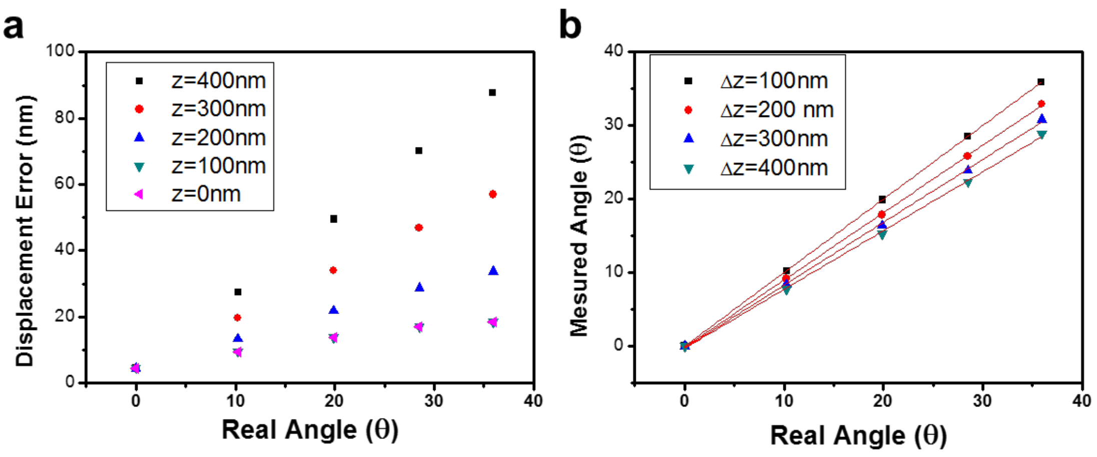


As shown in the figure (Figure a) the displacement error increases as the angle increases or the position of the image plane recedes from the center. The right graph (Figure b) shows the relation of the measured angle and the real angle, where the position of the image plane 1 is the center of the nanowire's motion. Note that up to Δz=100 nm, the measured angle almost same with the real angle, because the effects of the inclined nanowire are same in both image planes and vanish by calculating the difference of the displacement, $r_{2\_measured} - r_{1\_measured}$, to obtain angle.

For small angle, the measured angles are almost proportional to real angles. The proportionality constant depends on Δz, which is close to 1 in the vicinity of the center and deviates more from one as the image plane2 recedes from the center.

From the simulation, we confirmed that the effect of the DOF is related to both length and angle of the nanowire and the error caused by DOF can be overcome by using a longer nanowire, a short-wavelength illumination or by adding an error correction term as shown below.

The measured displacements are related to each other by following relation:

$$r_{2\_measured} = r_{1\_measured} + \Delta z\ \ tan(\theta \times w(\Delta z)) \quad \text{(S8)}$$

where $r_{2\_measured} =$ , $= r_{1\_measured}$, and $w(\Delta z)$ is the proportionality constant.

Using this relation, we can obtain the real angle $\theta$:

$$\theta = atan\left(\frac{r_{2\_measured} - r_{1\_measured})}{\Delta z} z\right) / w(\Delta z) \quad \text{(S9)}$$

Consequently, we obtain the real displacement of the nanowire:

$$r_{2\_real} \cong r_{1\_measured} + \Delta z\ \ tan(atan\left(\frac{r_{2\_measured} - r_{1\_measured})}{\Delta z} z\right) / w(\Delta z)) \quad \text{(S10)}$$

In this way, we check the angle distribution of the SNW for Fig.5 as shown below.

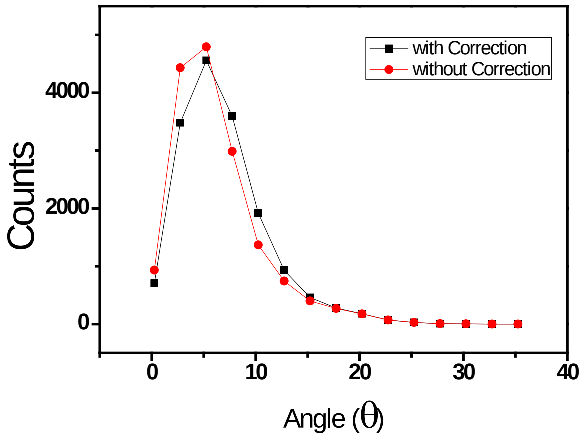


In this figure, we confirm that the angle without correction is underestimated. But the effect is not significant because the angle is not large value. Note that the effects vanish in the view of the power spectrum because the effects of the DOF multiply all displacements with same ratio.

## S6. The Angle measurement of the reference sample

To confirm the reliability of our method, we measured the angle of the reference nanowire by using our method and compared the results with the SEM image by following method.

The reference nanowires were obtained by PDMS spin coating and blading.

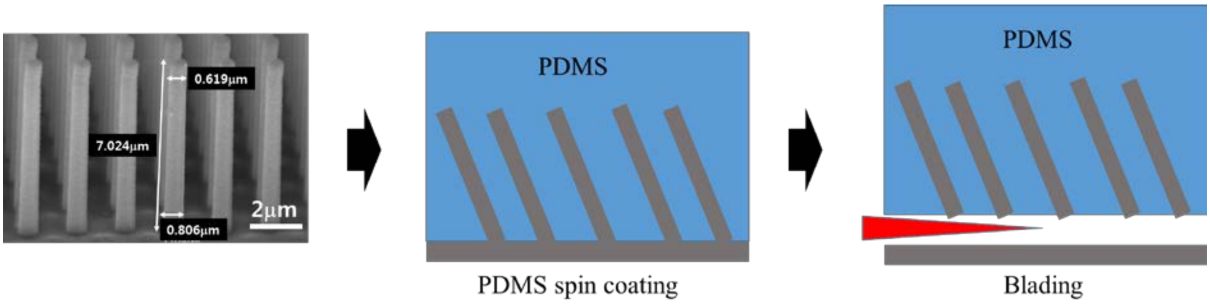


Consequently, we measured the angle of nanowires by using our method as shown in the following figure, where Δz is the distance between two image planes

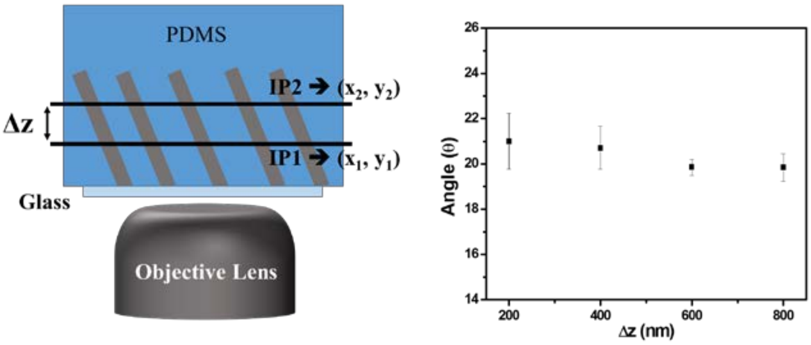


We measured the angle of the four nanowires with varying the distance between two image planes. The average angle is 20.36° and the standard deviation is 0.79°.

Finally, we confirmed that the results are well matched to the results which obtained from SEM image as shown below.

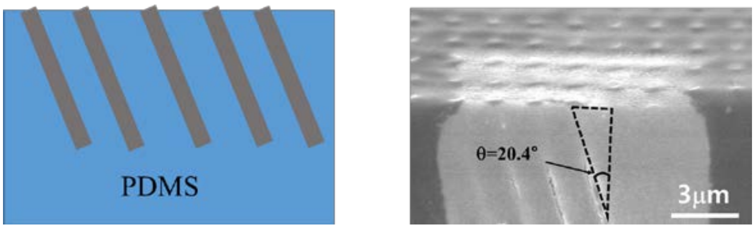

**S7. The cylindrical symmetry of the GaN nanowire**

The GaN nanowires with a <001> growth direction exhibit a hexagonal cross section. Our GaN nanowires were obtained by a top-down chemical etching method. Note that, in the experiment, the wavelengths of the illumination beam and trapping laser beam are much larger than the GaN nanowires' diameter. In this case, the assumption of the cylindrical symmetry is probably reasonable. In the top view image of the nanowire, the dark part is substrate and the bright part is the nanowire.

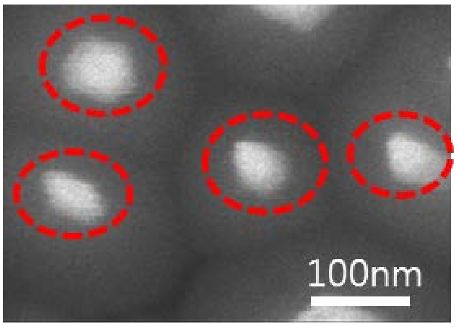


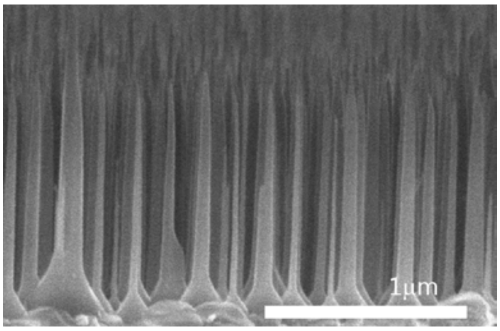

**S8. The image plane intersection ratio**

For tracking the nanowire's motion by using dual image planes, the nanowire should intersect both image planes simultaneously. In the experiment, the nanowire's edge can recede from the image planes due to its vertical translation and rotation (see the Figure below). To ensure that the nanowire intersects both image planes simultaneously, the space between two image planes should be no longer than 450 nm for 1 μm rod. The detail probability is shown figure b.

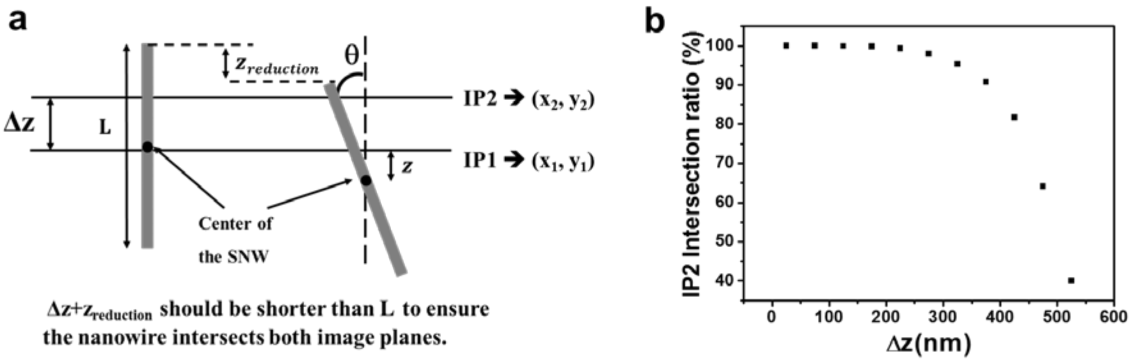

## S9. The asymmetry of the displacement along the z axis

In the figure. 3, we show the asymmetry of the displacement along the z axis. The origin of the effect is the geometrical asymmetry of the sample as shown below.

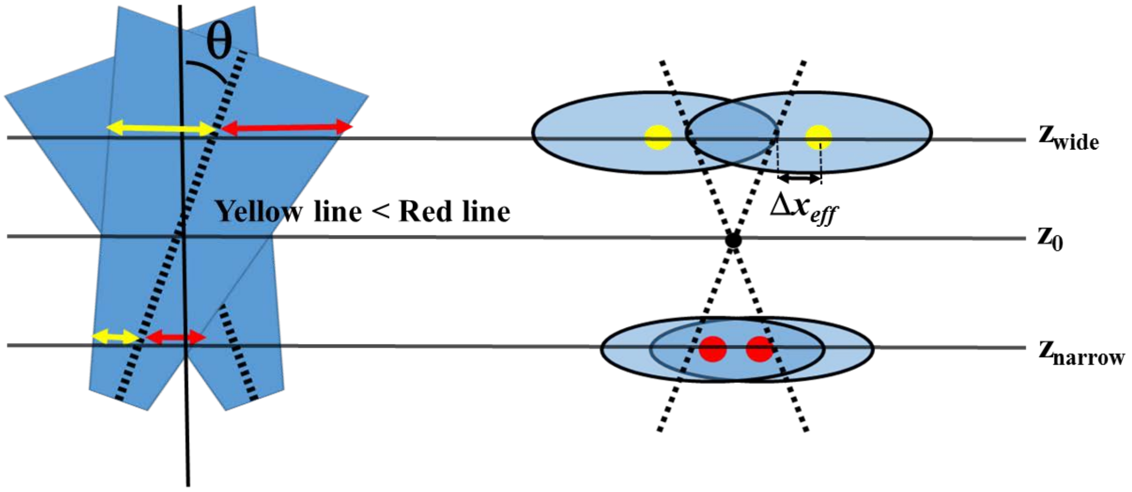


In the case of a cone shape particle, the center of the cross-section does not exist on the central axis of the cone, which is different in the case of a cylinder. The center of the cross-section at $z_{narrow}$ (see red dots) is inside the central axis (see dotted line) while the center of the cross-section at $z_{wide}$ (see yellow dots) is outside the central axis. Consequently, the x RMSDs at wide edge is larger than x RMSD at narrow edge. The $\Delta x_{eff}$ can be described as shown below:

$$\Delta x_{eff} = R(1+\cot\theta)^{\frac{1}{2}}\left(\frac{\cot\theta}{S+\ \cot\theta}\right) \quad \text{(S11)}$$

where R is the semi-major axis of the cross-section and S is the slope of the sample. In our cone shape sample, the $\Delta x_{eff}$ is -4.09 nm at the narrow edge and 6.43nm at the wide edge (minus sign means inward direction). It is well matched to the experimental results in the Figure 3 where the difference between the x RMSD values at top and bottom are 10 nm. Note that in the case of a cylinder, there is no effect because the center of the cross-section always exists on the

central axis.

**S10. The center position of the SNW**

Using dual image tracking within two image planes, the center position of the SNW, (xc, yc) can be obtained by adding the position measured by P1 and the variation caused by the incline of the SNW as shown in the figure below.

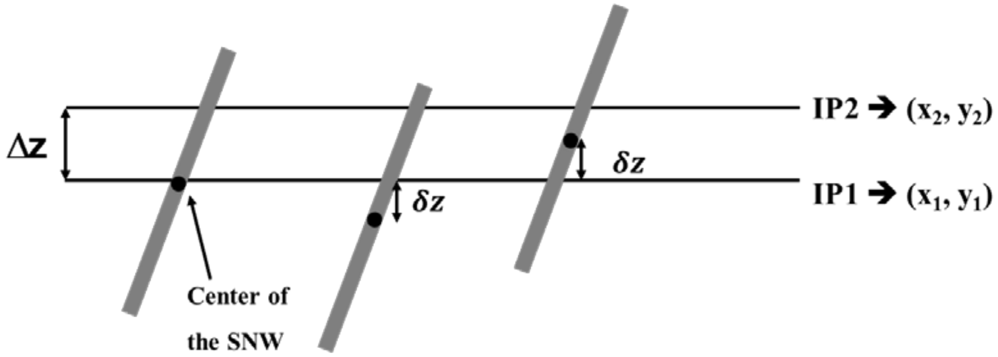


From this figure, equations for the center position can be presented in equation 2 and 3 of the main text.

## S11. Power spectrum analysis

The Brownian motion of a particle in an optical trap can be described as the Langevin equation with harmonic potential (Equation 6 of the main text). For now, we consider a dimensional Langevin equation for simplicity. If the momentum relaxation ti me is much shorter than the experimental time resolution, the second-order derivative term can be neglected and the equation becomes

$$\gamma \frac{\partial \mathrm{x}_i(t)}{dt} + k\mathrm{x}(t) = \sqrt{2k_B T\gamma}\zeta(t) \quad \text{(S12)}$$

If there is no potential caused by the optical tweezers, the Einstein equation can be obtained

$$D = \frac{k_B T}{\gamma} \quad \text{(S13)}$$

We Fourier transform x(t) and $\zeta(t)$ for time $T_{msr}$

$$\tilde{x} = \int_{-\frac{T_{msr}}{2}}^{\frac{T_{msr}}{2}} dt\, e^{i2\pi f t} x(t) \quad \text{(S14)}$$

By putting Equations (S13) and (S14) into Equation (S12), we can obtain

$$\tilde{x} = \frac{(2D)^{1/2}\tilde{\zeta}}{-i2\pi f + k/\gamma} \quad \text{(S15)}$$

The power spectrum $P_t$ is given by the Lorentzian form[3, 4]

$$P_t \equiv \langle \frac{|\tilde{\mathrm{x}}|^2}{T_{msr}} \rangle = \frac{D/2\pi^2}{f^2 + k/(2\pi\gamma)} \quad \text{(S16)}$$

The finite data acquisition rate results in the aliasing effect, and the power spectrum is modified to the aliased Lorentzian as shown below:[3, 4]

$$P_t \equiv \langle \frac{|\tilde{\mathrm{x}}|^2}{T_{msr}} \rangle = \frac{\Delta x^2/2\pi^2}{1 + c^2 - 2c\cos(2\pi f \Delta t)} \quad \text{(S17)}$$

where $c \equiv \exp(-\pi f_c/f_{Nyq})$, $f_{Nyq} \equiv f_{Sample}/2 = 256$, $\Delta x \equiv ((1-c^2)D/(2\pi f_c))^{1/2}$, $\Delta t \equiv 1/f_{Sample}$, and $f_{Sample}$ is the data acquisition rate. We obtained the power spectrum by averaging from 5 data set.

**S12. Friction constant**

If a rod moves along the axial direction, the rod will feel a hydrodynamic drag, which is parallel to the axial direction and is proportional to the friction constant $\gamma_{\parallel}$. In the other direction which is perpendicular to the axial direction, the friction constant is $\gamma_{\perp}$. Furthermore, we should consider the rotational motion. Because we can ignore the rotation around the axial axis for a thin cylindrical symmetric object, the rotational friction constant, $\gamma_{\mathrm{rot}}$, is enough. In general, the constants are not equal to each other and depend on the geometry of the sample as described as[5, 6]

$$\gamma_{\perp} = \frac{4\pi\eta L}{\ln P + \delta_{\perp}} \quad \text{(S18)}$$

$$\gamma_{\parallel} = \frac{2\pi\eta L}{\ln P + \delta_{\parallel}} \quad \text{(S19)}$$

$$\gamma_{\mathrm{rot}} = \frac{\pi\eta L^3}{3(\ln P + \delta_{\theta})} \quad \text{(S20)}$$

where $\eta$ is the water dynamical viscosity, $P$ is the aspect ratio defined as $P \equiv L/d$, and $\delta_i$ are end-effect corrections[7] as polynomial expression of $\nu=(\ln 2P)^{-1}$

$$\delta_{\perp} = 0.866 - 0.15\nu - 8.1\nu^2 + 18\nu^3 - 9\nu^4 \quad \text{(S21)}$$

$$\delta_{\parallel} = -0.114 - 0.15\nu - 13.5\nu^2 + 37\nu^3 - 22\nu^4 \quad \text{(S22)}$$

$$\delta_{\theta} = -0.446 - 0.2\nu - 16\nu^2 + 63\nu^3 - 62\nu^4 \quad \text{(S23)}$$

## S13. The angle distribution with varying laser power

We measured the motion of the nanowire in an optical trap with varying laser power, and analyzed the motion using the modified Langevin equation for $\Theta_i(t)$ under the condition $\sin\theta \approx \theta$. The higher power makes produced a the narrower angle distribution as expected. Even at the lowest laser power, most of the angles were smaller than 30°, at which the difference between the sine(sin30°=0.5) and the radians(0.524) yields only a 5% error.

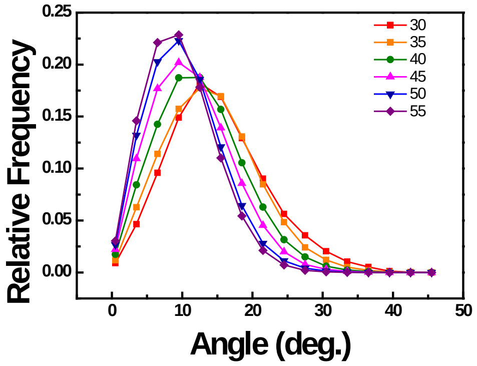

## S14. Angular displacements

We described the motion of the SNW in an optical trap as the angle $(\theta,\ \phi)$ with respect to the laboratory coordinate *xyz* axis (Figure 1-b). To describe the motion of the SNW with torque constant, the internal coordinates *x'y'z'* are much better than the laboratory coordinates. The corresponding relation between the two coordinates can be described as the tensor form $x_i' = R_{ij}(\phi',\theta')x_i$ where the matrix R is

$$R(\phi',\theta') = R_{X'}(\theta')R_Z(\phi') = \begin{pmatrix} 1 & 0 & 0 \\ 0 & \cos\theta' & \sin\theta' \\ 0 & -\sin\theta' & \cos\theta' \end{pmatrix}\begin{pmatrix} \cos\phi' & \sin\phi' & 0 \\ -\sin\phi' & \cos\phi' & 0 \\ 0 & 0 & 1 \end{pmatrix} =$$

$$\begin{pmatrix} \cos\phi' & \sin\phi' & 0 \\ -\cos\theta'\sin\phi' & \cos\theta'\cos\phi' & \sin\theta \\ \sin\theta'\sin\phi' & -\sin\theta'\cos\phi' & \cos\theta' \end{pmatrix} \quad \text{(S24)}$$

where $\phi'$ and $\theta'$ are the Euler angle with $\psi$, which is neglected for the cylindrical symmetric particles. As shown by the rotation matrix R, each of the components of *x'y'z'* are related to the lab coordinates *xyz*. The Langevin equation is very complex and almost useless. To simplify the analysis, we use the additional condition from our experimental data, $\sin\theta \approx \theta$ (Supplementary Information 13). In this case, the Euler angle $\theta'$ $\phi'$ is almost same as the angle $(\theta,\ \phi)$ with respect to the laboratory coordinate *xyz* axis. And the fully decomposed Langevin equation for the angular displacements, $\Theta_x = \theta\cos\phi$ and $\Theta_y = \theta\sin\phi$, can be obtained.

## S15. The specification of the illumination beam

In the experiment, we used the LED lamp (Thorlabs; M850L3, $\lambda_{MAX}$=850 nm) as the illumination beam. The FWHM is about 50 nm. The spectrum is shown below.

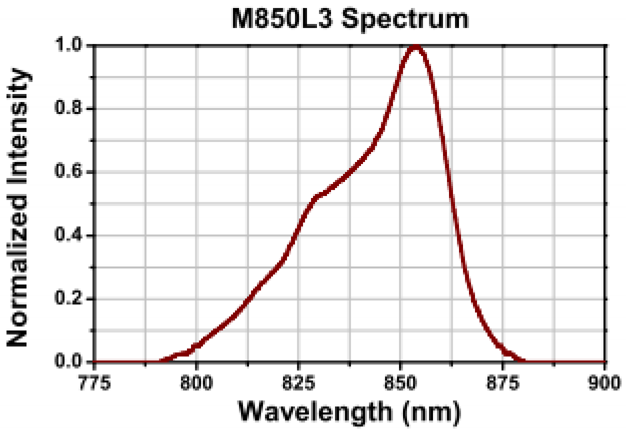


The more detail information exists in the website of Thorlabs.[8]

## Reference


1 Wagner, C., Stangner, T., Gutsche, C., Ueberschär, O. & Kremer, F. Optical tweezers setup with optical height detection and active height regulation under white light illumination. *Journal of Optics* **13**, 115302 (2011).

2 Sommargren, G. E. & Weaver, H. J. Diffraction of light by an opaque sphere. 1: Description and properties of the diffraction pattern. *Applied optics* **29**, 4646-4657 (1990).

3 Berg-Sørensen, K. & Flyvbjerg, H. Power spectrum analysis for optical tweezers. *Review of Scientific Instruments* **75**, 594-612 (2004).

4 Tolić-Nørrelykke, I. M., Berg-Sørensen, K. & Flyvbjerg, H. MatLab program for precision calibration of optical tweezers. *Computer physics communications* **159**, 225-240 (2004).

5 Broersma, S. Viscous force and torque constants for a cylinder. *The Journal of Chemical Physics* **74**, 6989-6990 (1981).

6 Tirado, M. M., Martinez, C. L. & de la Torre, J. G. Comparison of theories for the translational and rotational diffusion coefficients of rod-like macromolecules. Application to short DNA fragments. *The Journal of chemical physics* **81**, 2047-2052 (1984).

7 Marago, O. *et al.* Femtonewton force sensing with optically trapped nanotubes. *Nano Letters* **8**, 3211-3216 (2008).

8 Thorlabs https://www.thorlabs.com/thorproduct.cfm?partnumber=M850L3
